# Effects of increasing levels of whole Black Soldier Fly (*Hermetia illucens*) larvae in broiler rations on acceptance, nutrient and energy intakes and utilization, and growth performance of broilers

**DOI:** 10.1016/j.psj.2022.102202

**Published:** 2022-09-24

**Authors:** M.M. Seyedalmoosavi, M. Mielenz, S. Görs, P. Wolf, G. Daş, C.C. Metges

**Affiliations:** ⁎ Research Institute for Farm Animal Biology (FBN), Institute of Nutritional Physiology, Dummerstorf, Germany; † University of Rostock, Agricultural and Environmental Sciences, Chair of Nutrition Physiology and Animal Nutrition, Rostock, Germany

**Keywords:** chicken, edible environmental enrichment, feed preference, insect, whole larvae

## Abstract

Meal of black soldier fly larvae (**BSFL**), which requires extraction of protein and fat, is a novel protein source for poultry, while unprocessed whole BSFL could even directly be fed to chickens. Newly hatched Ross-308 chicks (n = 252) received whole BSFL at 10% (L10), 20% (L20), or 30% (L30) of voluntary feed intake (**FI**) of control chickens (**CON**) that received no BSFL but only age-specific diets (n = 63 birds / group) for 42 days (**d**). Acceptance and nutrient and energy intake of birds by BSFL and FI were calculated. Plasma metabolites were measured using an automatic enzymatic analyzer and immunoglobulins with ELISA. Depending on the variable, data were analyzed using ANOVA or repeated measures ANOVA to address treatment, time and interaction effects. Birds consumed all offered larvae. With the exception of d1, time spent by birds eating their daily portion of larvae (TSL, min/pen) did not differ among the larvae supply groups (*P* = 0.982). The L10 had a higher larvae eating rate (**LER**) that is, speed of larvae intake than did L20 and L30 (*P* < 0.05), implying increased competition for less available BSFL. The ratio of LER to feed eating rate (**FER**) was greater than 50 fold change difference (**FCD**), indicating a strong interest of chickens in BSFL over regular feed. Whole BSFL intake up to 30% of voluntary FI did not adversely affect broiler growth (*P* > 0.05). The L30 had lower total dry matter and metabolizable energy intakes (*P* < 0.05), although total fat intake was higher in L30 than in CON (*P* < 0.05). Compared with CON, 30% whole BSFL increased dietary protein-to-energy ratios, plasma uric acid and serum alkaline phosphatase concentrations (*P* < 0.05). We conclude that whole BSFL can be included in broiler rations up to 20% without negatively affecting growth performance and nutrient conversion efficiency, whereas a higher proportion is associated with lower protein utilization efficiency, possibly due to lower total energy intake.

## INTRODUCTION

Black soldier fly larvae (**BSFL**; *Hermetia illucens*) meal has been suggested as a sustainable alternative protein source to soybean meal (Maurer et al., 2016; Schiavone et al., 2017; Cutrignelli et al., 2018; El-Hack et al., 2020; de Souza Vilela et al., 2021). BSFL contain approximately 31 to 45% crude protein (**CP**) in dry matter (**DM**), and are rich in minerals such as Ca and P, implying an important ingredient for chicken diets (Maurer et al., 2016; Bava et al., 2019). The inclusion of partially defatted BSFL meal in broiler diets at 10% increased feed intake (**FI**) and average daily gain and improved feed conversion efficiency (Attivi et al., 2020). The suitability of BSFL-meal up to 20% in broiler diets was confirmed by higher body weight (**BW**) in the grower and finisher phases (de Souza Vilela et al., 2021). Recent studies have further revealed that the inclusion of BSFL meal in broiler diets may enhance immune functions, likely due to bioactive compounds of BSFL such as chitin and lauric acid (Fariz Zahir Ali et al., 2019; Dörper et al., 2020). The inclusion of BSFL meal in broiler diets however implies the extraction of protein and fat from the larvae, which requires expensive feed processing technology. In addition, drying of BSFL may result in lower availability and ileal digestibility of certain amino acids in broiler diets due to the Maillard reaction (Ruhnke et al., 2018), and may influence the organoleptic characteristics for the birds (Moula et al., 2018). In contrast to the meal form, less is known about the inclusion of unprocessed whole BSFL in poultry rations on acceptance, nutrient intake and utilization, performance and health of the birds. Chickens are excellent foragers of insects as these are among their natural feed sources (Star et al., 2020). Feeding experiments indicated that diets containing insects are highly interesting for poultry species (Moula et al., 2018; Nascimento Filho et al., 2020; Star et al., 2020; Tahamtani et al., 2021). The use of whole BSFL as feed for poultry may be particularly important in organic farming and low-input systems (e.g., local farming with less feed processing and transportation), and where insect production could be integrated into production cycles (e.g., insects farming with locally available organic residues as feed substrate) (Nyakeri et al., 2017). In this context, it has been reported that BW of chickens fed a standard diet supplemented with 8% whole defrozen larvae were higher than in control chickens (Moula et al., 2018). In addition, inclusion of 5 and 10% live BSFL in broiler rations increased activity and improved leg health without adverse effects on broiler performance (Ipema et al., 2020b; Bellezza Oddon et al., 2021). Recently, Tahamtani et al. (2021) fed laying hens with 0, 10, 20% of the daily DM intake or ad libitum with live BSFL and found no difference in BW of hens given BSFL up to 20%, whereas ad libitum BSFL fed hens were heavier and consumed more protein, fat and energy than control hens. However, there are no data to show how a proportion of more than 10% whole BSFL in broiler rations affects acceptance, nutrient intakes, bird performance and health. It is well known that the nutrient composition and form of broiler diets impact on their energy intake (Latshaw, 2008). Broilers can adjust their feed intake (**FI**) in response to alterations of certain dietary factors such as energy level (Hu et al., 2021), amino acid balance (Ferket and Gernat, 2006), fiber content (Jha and Mishra, 2021), and mineral balance (Delezie et al., 2015). Nutrient, moisture, and energy contents of BSFL and standard broiler diets differ greatly (e.g., see Table 1). Therefore, we hypothesized that the provision of whole BSFL on top of balanced broiler rations, particularly at high levels, induces trade-offs in nutrient and energy intake from regular feed and BSFL, ultimately resulting in imbalances and lower nutrient efficiency and impaired growth performance. Consequently, the objective of this study was to first investigate the interest of broiler chickens in consuming whole BSFL and then to determine the extent to which the inclusion of whole BSFL in broiler rations influences nutrient and energy intakes, utilization efficiency, as well as health statutes, growth performance, selected blood metabolites and immunity of the birds.

**Table 1:** Ingredients and analysed chemical composition of the age-specific diets and black soldier fly larvae (BSFL) offered to broilers during the experimental period.

	Basal diets			
	Starter (d 1–14)	Grower (d 15–28)	Finisher (d 29–42)	BSFL (d 1–42)
Ingredients, % as fed				
Soybean meal 48%	36.0	34.0	26.5	-
Wheat	31.0	28.0	35.0	-
Maize	21.5	28.0	28.0	-
Barley	5.0	4.0	5.0	-
Linseed oil	3.0	3.0	3.0	-
Vitamin-mineral premix^1^	2.5	2.5	2.5	-
Oyster shells	1.0	0.5	0	-
Whole black solider fly larvae	-	-	-	100
Chemical analysis, g/kg DM				
Dry matter	893	891	892	312
Crude ash	61.6	48.3	44.8	80.4
Crude protein^2^	228	218	203	435
Crude fat	47.0	47.1	42.6	277.5
Crude fibre	24.6	25.8	34.8	72.7
Starch^3^	463.6	508.4	516.8	14.3
Total sugar (calculated as sucrose)	43.67	46.02	42.60	2.20
NDF	118	114	110	121
ADF	41.4	43.8	38.1	77.78
ADL	n.d.	n.d.	n.d.	4.42
Chitin*	n.d.	n.d.	n.d.	73.35
ME, MJ/kg DM	13.4	14.0	13.8	16.5
Minerals, g/kg DM				
Calcium	12.1	7.6	6.1	18.1
Phosphorus	6.6	5.2	5.2	9.3
Magnesium	2.1	1.9	2.0	3.9
Sodium	1.6	1.0	1.2	1.3
Potassium	10.2	9.0	8.9	12.8
Iron	0.024	0.019	0.022	0.012
Manganese	0.011	0.084	0.011	0.021
Copper	0.017	0.014	0.019	0.010
Zinc	0.097	0.074	0.093	0.013

Abbreviations: ADF, acid detergent fiber; ADL, acid detergent lignin; NDF, neutral detergent fiber; n.d., not determined.

⁎ Calculated based on Hahn et al. (Hahn, et al., 2018; i.e., Chitin = ADF - ADL).

1 Amount of vitamin and minerals provided through premix per kg of feed are as following; Vit. A (from vitamin A acetate), 10,000 IU; Vit. D3, 2,000 IU; Vit. E (from DL-α-tocopherol acetate), 20 mg; Vit. K3, 3 mg; Vit. B1, 1 mg; Vit. B2, 6 mg; Vit. B6, 3 mg; Vit. B12, 30 mcg; Niacin, 30 mg; Pantothenic acid, 10.8 mg; Folic acid, 0.4 mg; Biotin 24, mcg; Cholin, 300 mg; Fe, 55 mg; Cu, 18 mg; Zn, 80 mg; Mn, 93 mg; I, 0.66 mg; Se, 0.34 mg; Co, 0.05 mg; Phytase, 250 FTU.

2 Crude protein content of BSFL might be overestimated due to the high non-protein content (e.g. chitin) (Shumo, et al., 2019). For amino acid composition see Supplementary Table 1.

3 For BSFL it is glycogen.

## MATERIALS AND METHODS

A feeding experiment was conducted over 6 weeks (**wk**). Animal care and handling, stunning and slaughtering of the birds were performed by trained and authorized staff. The feeding experiment was registered under A.Z. 202022_70_A28_anz.

### Animals and Management

A total of 252 newly hatched chicks (Ross 308) was obtained from a commercial hatchery and housed at the experimental poultry facility at the Research Institute for Farm Animal Biology (**FBN**), Dummerstorf, Germany. The chicks were weighed at arrival and randomly allocated to one of 24 pens (n = 10–11 chicks / pen) in 4 adjacent rooms of the facility. Pens in each room (n = 6) were separated from each other with solid panels. Each pen was equipped with a feeder, a line of drinking nipples with cups, and a deep layer of wood shavings as litter material. Throughout the experiment, birds in different rooms were kept under the same environmental conditions. Climate conditions in the rooms were automatically controlled based on recommendations of the Aviagen Ross broiler handbook (Aviagen, 2018) by a ventilation and heating system, ensuring uniform temperature, light and aeration conditions across the pens within and between the 4 experimental rooms. Ambient temperature at the start of the experiment was 33°C and this was gradually decreased to 21°C at wk 6, whereas humidity was gradually increased from 37 to 70% until wk 6. The light program included a 21-hours (**h**) light (30–40 Lux) and a 3-h dark period during the first 3 days (**d**). By d 4, lighting was changed to 18 h of light (15–20 Lux) and 6h of darkness until the end of the experiment.

### Experimental Design

A completely randomized design with 4 treatments was used in this study. All birds received the same basal diet, designed to meet or exceed age-specific nutrient requirements of broilers in 3 phases, that is starter (d 0–14), grower (d 15–28), and finisher (d 29–42) diets (Table 1; Aviagen, 2019). Equal number of pens (n = 6 per group) and birds (n = 63 per group, n = 10–11 per pen) were randomly allocated to each of 4 dietary treatments in four adjacent rooms. Each of the 4 treatment groups was represented in each of the 4 rooms with one or 2 pen-replications with further randomization for the position of the pens/treatment groups in the rooms. Broilers in the control group (**CON**, n = 63 birds in 6 pens) received the age-specific basal diet ad libitum, and had no access to BSFL. Birds in the remaining 18 pens received defrozen whole BSFL in addition to the ad libitum offered basal diet at increasing levels, that is, 10, 20, or 30% of the FI of CON birds (hereafter referred to as groups **L10** (n = 63), **L20** (n = 63), and **L30** (n = 63), respectively). Except for the first day of life, the daily amount of BSFL to be fed to the broilers in the L10 to L30 groups was calculated based on the FI of the CON birds on the previous day. At the first day of life, FI of broiler birds from previous experiments was used as a reference. Whole BSFL were purchased from Hermetia Deutschland GmbH & Co. KG, Baruth/Mark, Germany. All the larvae used in this experiment originated from the same rearing batch. Analyzed chemical composition of the BSFL is provided in Table 1. As soon as the live larvae were received, they were snap frozen using liquid nitrogen and stored at −20°C until fed to broilers. Approximately 12 h before feeding larvae to broilers, the larvae were stored in a refrigerator (4°C) to thaw and then kept at room temperature for weighing of the daily portions for broilers in the L10–L30 groups. Age-specific diets were in the form of regular feed that was coarsely ground and not pelleted. Ingredients and analyzed chemical composition of the age-specific diets are provided in Table 1. The diets were purchased from a commercial feed producer (Ceravis AG, Rendsburg, Germany).

### Larvae Provision and Time Records

In order to assess acceptance and interest of chickens in BSFL, we recorded the time broilers spent eating larvae and calculated a larvae eating rate. Birds in larvae supply groups, that is, L10, L20, and L30 received defrozen BSFL at the same time each day (by 07:30 h). For this purpose, the defrozen larvae to be given to the birds of a pen were weighed and placed on a feeding plate. The plate was placed on the ground of the recipient pen, and the start time of larva eating by the birds was recorded. The pens were observed frequently, and the time when there were no more larvae left on the plate was recorded with a precision of 1 min. The time spent eating BSFL (**TSL**, min/pen) was then calculated in min (i.e., end time – start time) for each day. The BSFL eating rate (**LER**, g/min) of the birds, that is, the amount of BSFL eaten per min was calculated. The LER was also adjusted for the BW of the birds in the pen (**LER_BW**), that is, the amount of BSFL eaten per min and kg BW of chickens in a pen (g BSFL / kg BW^−1^ min^−1^). The LER_BW was estimated only for the last d of each wk, where the corresponding BW for that wk was measured. Similar to LER, a theoretical feed eating rate (**FER**) was also calculated. For this purpose, the daily amount of feed consumed by the birds of a pen was divided by the duration of the light period of the day (i.e., g feed / daily light period in min). The FER was also adjusted for BW (i.e., **FER_BW**: g feed / kg BW^−1^ min^−1 in light period^), using corresponding FI and BW data on the last d of each wk. Finally, the ratio of BSFL eating rate to feed eating rate was calculated as fold change differences (**FCD**, i.e., LER:FER) without and with consideration of BW (**FCD_BW**). Throughout the study, larvae provisioning and time records were kept by the same person.

### Feed Intake and Growth Performance

Pen based daily FI from the previous day was measured in the mornings, and average daily or weekly total FI per bird was then calculated. The weekly total fresh matter intake (**FMI**; the sum of feed and larval intake) and the resulting dry matter intake (**DMI**) per average bird of each pen were calculated. Based on the amounts of feed and BSFL intakes, and the nutrient and energy contents of the diets and BSFL, pen based nutrient (e.g., protein, fat) and metabolizable energy (**ME**) intakes were calculated for an average bird. The growth performance of the broilers was evaluated throughout the experimental period. The pen based average BW, FI, FMI, DMI, feed conversion ratio (**FCR**) with consideration of either FMI (i.e., FCR-1: g FMI per g BW gain) or DMI (i.e., FCR-2: g DMI per g BW gain), protein conversion ratio (**PCR**: g protein intake to gain 100 g BW) and energy conversion ratio (**ECR**: MJ ME intake to gain 100 g BW) of the birds were calculated on a weekly basis. To assess the homogeneity of growth of birds in a pen, a weekly coefficient of variation (**CV**) of BW was calculated for each pen.

### Foot and Leg Health and Litter Moisture

Footpad dermatitis (**FPD**) and hock burn (**HB**) assessments were performed prior to slaughter on d 28 and d 42 using the Welfare Quality assessment protocol for poultry (Welfare-Quality, 2009). If feet were dirty, they were carefully cleaned with a damp cloth before scoring; only the central plantar area was scored, and signs of foot pad lesions were recorded on a 5-point scale, where 0 indicated no lesion. Litter samples (∼100 g) to measure moisture were collected from each pen on d 42. Samples were oven-dried at 103°C until they reached a constant weight.

### Chemical Analysis of Diets and BSFL

During the experiment, feed and larval samples were collected regularly and stored at −20°C for chemical analyses. At the end of the experiment, all of the subsamples were pooled by feed type (e.g., starter, grower, and finisher) and representative samples were analyzed for their nutrient contents. Larvae and feed samples were analyzed for DM content, crude ash, CP, crude fat, starch, crude fibre (**CF**), neutral detergent fiber (**NDF**), acid detergent fiber (**ADF**), and selected macro- and trace minerals (Table 1) by the accredited feed laboratory of Landwirtschaftliche Untersuchungs-und Forschungsanstalt, LMS Agrarberatung GmbH (Rostock, Germany) using standard methods (Naumann et al., 1997). The ME contents of feed and BSFL were then estimated using the equation, ME, MJ/kg DM = [(g CP × 0·01551) + (g CL × 0·03431) + (g starch × 0·01669) + (g sucrose × 0·01301)]. For BSFL, acid detergent lignin (**ADL**) content was additionally determined to estimate chitin (i.e., ADF - ADL) content (Hahn et al., 2018). Table 1 presents the ingredients and chemical compositions of the age-specific basal diets, and summarizes the nutrient composition of BSFL. Age-specific basal diets and drinking water were provided ad libitum throughout the experimental period.

The amino acid (**AA**) compositions of the diets and larvae samples were determined using high performance liquid chromatography (**HPLC**) (1200/1260 Infinity II series, Agilent Technology, Waldbronn, Germany) (Kuhla et al., 2010) after acidic hydrolysis of samples using a 250 × 4.6 Gemini 5 μm reversed-phase C18 110 Å column protected with a 4 × 3 pre-column (Phenomenex, Germany). Five mg of lyophilized ground sample was suspended in 2 ml of 6 M HCl. After addition of 50 µL of ascorbic acid (16 mg/mL ultrapure water), oxygen was removed from the suspension with a strong N_2_ flow for 1 min, and then the sample was heated for 22 h at 110 °C. The hydrolysate was dried at 60°C with a constant N_2_ flow and then re-suspended in 2 ml of 0.1 M HCl. The suspension was then centrifuged at 1,573 × *g* at 4°C for 20 min. For AA analysis the supernatant was diluted 1/10 with ultrapure water. The AA chromatograms were integrated with the OpenLab ChemStation software (Agilent Technologies, Waldbronn, Germany) and the AA concentrations were calculated based on a calibration with a standard AA mixture (A9906, Sigma-Aldrich/Merck, Darmstadt, Germany). The AA compositions (%) of the diets and BSFL are summarized in Supplementary Table 1.

### Blood Metabolites and Immunoglobulin Isotypes

In the end of experimental wk 4 and wk 6, two birds per pen (n = 48 / time point) were randomly chosen, weighed, and slaughtered after electrical stunning. From each bird, slaughter blood was collected to obtain serum and plasma. For serum collection blood samples were kept at room temperature for approximately 1 h to allow for clotting, and were then centrifuged (2,500 × *g* for 20 min at 4°C). Serum was harvested and stored in Eppendorf vials (Sarstedt AG & Co., Nümbrecht, Germany) at −20°C until analysis of alkaline phosphatase (**ALP**) activity using an automatic enzymatic analyzer (ABX Pentra 400, Horiba Medical, Montpellier, France) with a commercial kit (ALP Kit No. A11A01626). Plasma was harvested after centrifugation of K3-EDTA-coated evacuated tubes (Sarstedt AG & Co.). Plasma albumin, total protein and uric acid (**UA**) concentrations were analyzed with ABX Pentra 400 using commercial kits [albumin: Kit No. A11A01664, total protein: Kit No. 553-412, uric acid: Kit No. LT-UR0010 (MTI diagnostics, Idstein, Germany)]. Globulin concentration was calculated as total protein minus albumin. Commercial ELISA kits (IgY: Kit No. E30-104; IgM: Kit No. E30-103; IgA: Kit No. E30-102; Bethyl Laboratories, Inc, Montgomery, TX) were used to analyze immunoglobulin isotype concentration (IgY, IgM, IgA) in plasma samples. The intra-assay CV and inter-assay CV of Ig analysis ranged between 5.0–7.6% and 7.7–10.4%, respectively.

### Statistical Analysis

The pen was considered the experimental unit for all pen-based measurements, for example, daily or weekly feed intake, larvae intake and nutrient intakes, time spent eating larvae, larval and feed eating rates, and BW. Weekly measured pen data for growth parameters (e.g., BW), daily nutrient and energy intake (e.g., weekly FI, protein intake, energy intake), and the corresponding weekly nutrient and energy conversion indices calculated for FCR, PCR, and ECR were analyzed using a linear mixed model (PROC MIXED) in SAS (version 9.4; SAS Institute Inc., Cary, NC). The statistical model included fixed effects of the treatment group (1–4), wk (1–6) and treatment group by week interaction. The blocking effect of rooms (1–4) was also included in the model. Pen (n = 24) was considered as repeatedly measured subject over time, and was implemented in the statistical model. For daily measured or calculated variables (e.g., time spent larvae eating, larval and feed eating rates), the abovementioned model was used with day (1–42) instead of week effects. For the single-point measurements (e.g., blood metabolites and IgY isotypes) the experimental unit was a bird sampled at slaughterhouse (N = 96). Thus, data related to the parameters measured on individual birds were analyzed by the general linear model procedure of SAS (PROC GLM). The statistical model included fixed effects of treatment group (1–4) and slaughter week (4, 6), interaction term, and the blocking effects of room and pens. Group differences were separated by Tukey-Kramer test at *P* < 0.05. The SLICE statement of PROC MIXED of SAS was used to conduct partitioned analyses of the LSM for interactions between treatment by day (or week) when required. The significance level was preset at *P* < 0.05, and a tendency was declared at 0.05 < *P* ≤ 0.10. Values are presented as LSM with their SE.

A principal component analysis (**PCA**) was conducted using JMP statistical software V.15 (SAS Institute) to investigate potential trade-offs in nutrient intakes due to different levels of BSFL in the broiler ration, and identify relevant nutrients as potential driving forces that are principally associated with differentiation among the groups in nutrient and energy intakes. For this purpose we selected 5 representative nutrients to address energy (i.e., crude fat and starch intakes), protein (CP intake), dietary fiber (NDF intake), mineral (crude ash) intakes, and conducted a PCA using the weekly recorded data of the corresponding nutrients collectively.

## RESULTS

All chickens appeared to be healthy, and no bird died during the experiment. The overall average FPD and HB scores were 0.0 and 0.02, respectively. Because of FPD and HB were absent or extremely low no statistical comparison of the groups was performed. The DM content of the litter ranged from 78 to 81%, and did not differ among the 4 groups (*P* > 0.05; further data not shown).

### Black Soldier Fly Larvae and Feed Intakes

**Figure 1:**
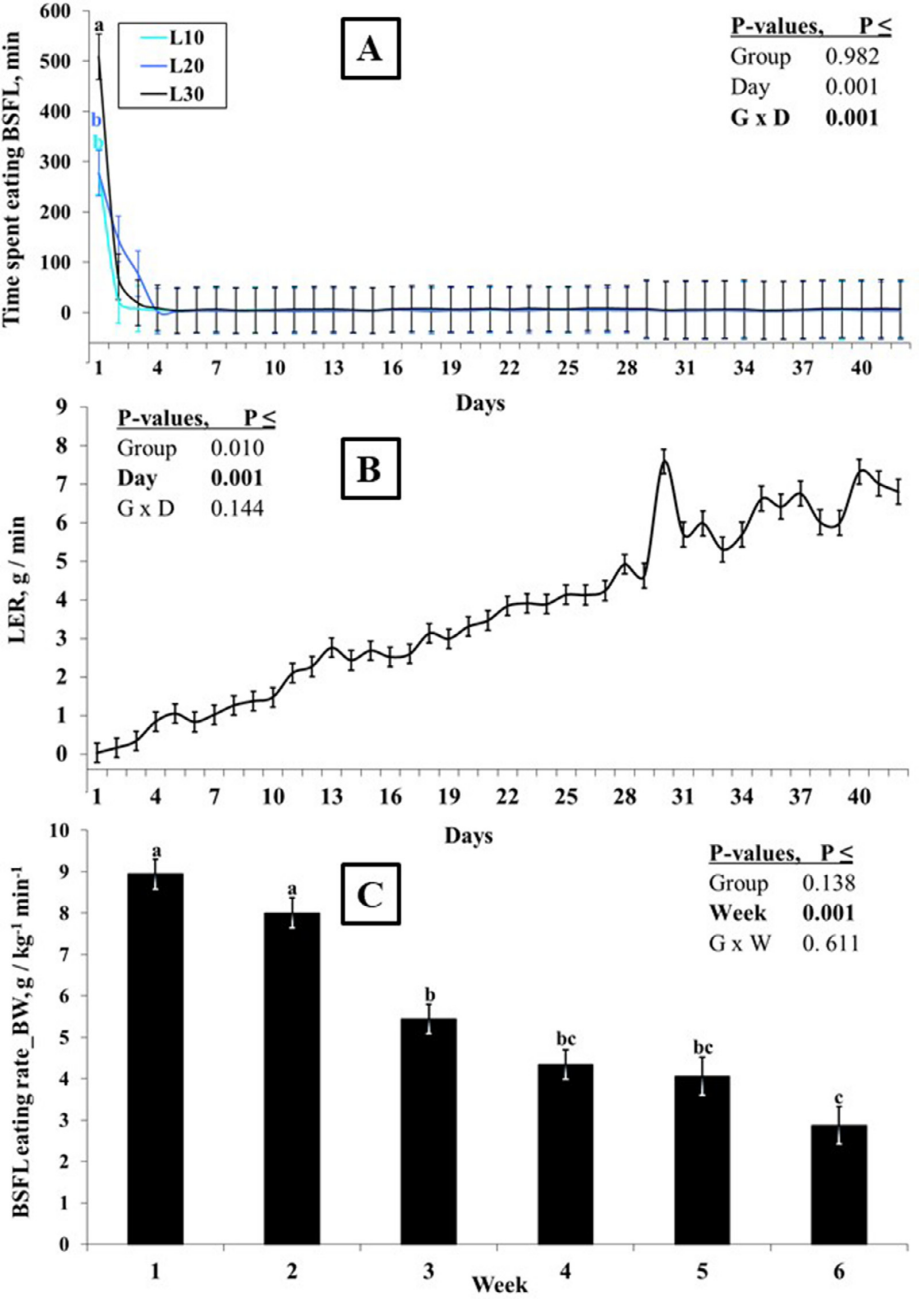
Time spent eating whole black soldier fly larvae (BSFL) (TSL) (A), larvae eating rate (LER) (B) and body weight adjusted LER (C) in broilers offered BSFL at 10% (L10), 20% (L20), or 30% (L30) of daily feed intake of control chickens. Values are LSM with their SE. a-c: Values denoted with different letters within each panel differ significantly (Tukey, *P* < 0.05).

With the exception of the first 3 d, the daily pre-determined portions of BSFL offered to the birds were consumed fully within a few min in all 3 BSFL groups (Figure 1A). For instance, on d1 L30 birds spent 509 min to consume their portion of BSFL, whereas it took only 7 min to consume BSFL equivalent to 30% of the CON FI on d 42. The average TSL ranged between 11.3 and 20.5 min in L10 to L30 groups with no group difference (*P* = 0.982; Table 2) expect for the first d (*P* < 0.05; Figure 1A). On the first d, L30 birds spent more time (*P* < 0.05) to consume 30% of CON feed intake as BSFL than birds in the L10 and L20 groups. In the remaining 41 d, the TSL was similar among the 3 groups regardless of the amount of BSFL offered to the birds (*P* > 0.05). The LER depended on both the amount of BSFL offered to the birds (*P* = 0.010; Table 2) and experimental d (*P* < 0.001; Figure 1B). The L10 birds consumed their portion of BSFL at a higher rate (LER = 3.97 g/min) (*P* < 0.05) than the L20 and L30 birds (Table 2). The average total LER across the 3 groups increased linearly by more than 200-fold from the first day (0.03 g/min) to the last day (6.8 g/min) of the experiment (Figure 1B). When corrected for the increasing bird BW over time (i.e., LER_BW), a linearly decreasing pattern of BSFL eating rate was observed (*P* < 0.001; Figure 1C), which nevertheless showed no dependence on the amount of BSFL offered to the birds (*P* = 0.138; Table 2). The LER_BW showed a linear decrease of two thirds from approximately 9 to 3 g/kg per min. (Figure 1C).

**Table 2:** Impact of BSFL provision at increasing dietary levels on feed and BSFL eating rates in relation to time and body weight development of broilers.

	Dietary treatment groups^1^					*P*-values^2^, ≤		
	CON	L10	L20	L30	SE	G	W	G × W
BSFL intake, g *(N=126, i.e., 3G* × *42d)*	n.a.	9.1	1.2	27.3	n.a.	n.a.	n.a.	n.a.
TSL, min *(N=756, i.e., 3G* × *42d* × *6 pen)*	n.a.	11.3	15.6	20.5	33.65	0.982	0.001	0.001
LER, g / min *(N=756, i.e., 3G* × *42d* × *6 pen)*	n.a.	3.97^a^	3.59^b^	3.56^b^	0.085	0.010	0.001	0.144
LER_BW, g / kg^−1^ min^−1^ *(N=108, i.e., 3G* × *6wk* × *6 pen)*	n.a.	6.09	5.25	5.49	0.276	0.138	0.001	0.611
Feed intake, g/day *(N=1,008, i.e., 4G* × *42d* × *6 pen)*	94.1^a^	84.9^b^	84.5^b^	71.9^c^	1.16	0.001	0.001	0.001
FER, g / min in light period *(N=1,008, i.e., 4G* × *42d* × *6 pen)*	0.087^a^	0.079^b^	0.078^b^	0.066^c^	0.0011	0.001	0.001	0.001
FER_BW, g / kg^−1^ min^−1 in light period^ *(N=144, i.e., 4G* × *6wk* × *6 pen)*	0.112^a^^†^	0.105^ab^^†^	0.104^b^	0.096^c^	0.0018	0.001	0.001	0.001
FCD (LER:FER) *(N=108, i.e., 3G* × *6wk* × *6 pen)*	n.a.	55.4	50.1	56.6	2.58	0.195	0.018	0.386
FCD (LER:FER)_BW *(N=108, i.e., 3G* × *6wk* × *6 pen)*	n.a.	55.4	50.1	56.6	2.71	0.222	0.020	0.371

Abbreviations: FER, theoretical feed eating rate; FER_BW, theoretical feed eating rate corrected for kg BW; FCD, fold change difference in ratio of LER:FER; FCD_BW, FCD corrected for BW; LER, BSFL eating rate of chickens; LER_BW, BSFL eating rate of chickens adjusted per kg BW; TSL, time spent eating BSFL.

Sample size (N) and its calculation together with further information on pen replicates and time dimension are provided beneath of each variable in *italics*.

a-c Treatment groups denoted with different letters differ significantly (Tukey, *P* < 0.05). The sign (†) indicate tendency to differ (Tukey, 0.05 < *P* ≤ 0.10). Data are presented as LSEMANS and their SE.

1 Dietary treatments: ad-libitum feed without access to BSFL (CON), or with BSFL amounting to 10% (L10), 20% (L20), or 30% (L30) of the feed intake of CON birds. Total number of observations used for statistical analyses, N = 144 (4 treatments each with 6 replicate pens repeatedly measured over 6 weeks); Number of birds, n = 63 per treatment.

2 G = Group effect, W = time effect (week or day depending on the variable), G × W = group by time interaction.

**Figure 2:**
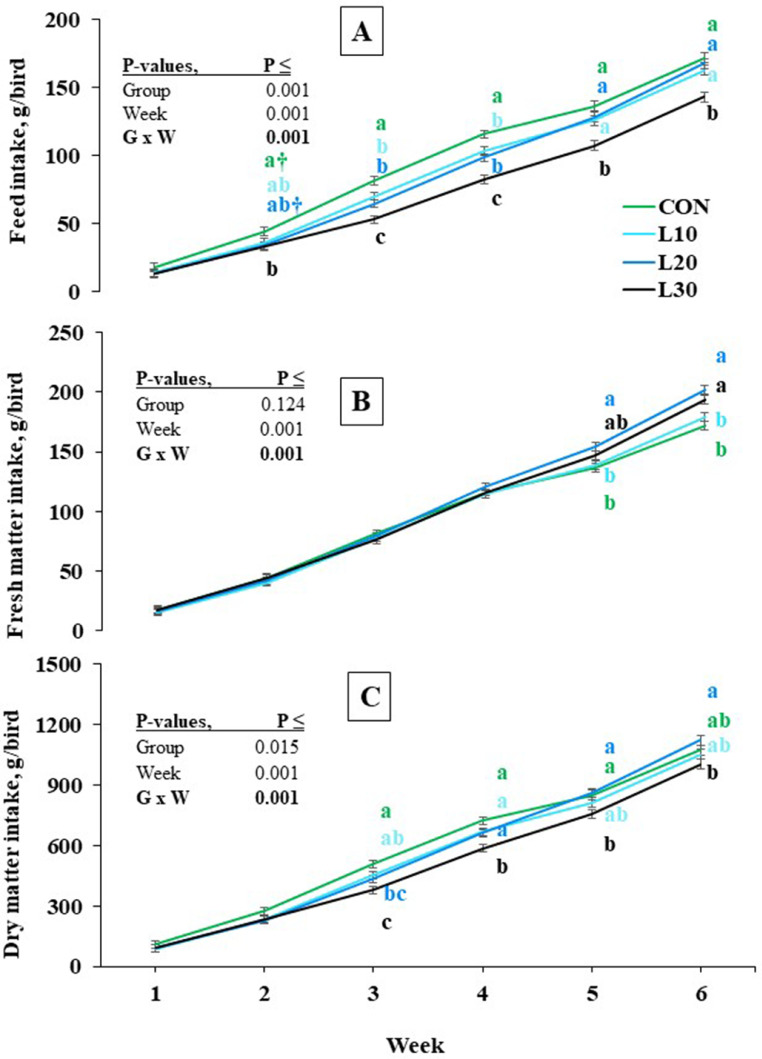
Effects of increasing levels of whole black soldier fly larvae in broiler rations on average feed intake (A), fresh matter intake (feed plus larvae) (B), and dry matter intake (feed plus larvae) (C) during the experimental weeks. Values are LSM with their SE. a-c: Values denoted with different letters at the same point within each panel differ significantly (Tukey, *P* < 0.05). The symbol † indicates a tendency of two treatments to differ (Tukey, 0.05 < *P* ≤ 0.10). The symbol † indicates a tendency of two treatments to differ (Tukey, 0.05 < *P* ≤ 0.10).

Provision of BSFL to broilers reduced (*P* < 0.001; Table 2) voluntary FI in both L10 and L20, and did more so strongly in L30 compared with CON in a time-dependent manner (*P* < 0.05; Figure 2A). The L30 group had a lower FI compared with CON already at wk 2, and this difference remained constant until the end of the experiment (*P* < 0.05; Figure 2A). The FI in L10 and L20 was lower than that of CON at wk 3 and 4 (*P* < 0.05). Until wk 5 of the experiment, there was no difference in FMI between groups (*P* > 0.05; Figure 2B). At wk 6, both L30 and L20 birds consumed a higher amount of FM than CON and L10 birds (*P* < 0.05). In contrast to L10 and L20 groups, birds offered BSFL at 30% of the CON intake had an impaired DM intake (*P* < 0.05; Table 3), as early as wk 3 (Figure 2C).

**Table 3:** Effects of increasing levels of whole black soldier fly larvae in broiler rations on fresh and dry matter intakes, nutrient and energy intakes, growth performance and nutrient and energy conversion ratios of broilers.

	Dietary treatment groups^1^					*P*-values^2^, ≤		
	CON	L10	L20	L30	SE	G	W	G × W
Nutrient and energy intakes								
Fresh matter, g / wk	662.3	661.2	718.3	694.3	17.95	0.124	0.001	0.001
Dry matter, g / wk	590.6^a^	552.7^ab^	566.9^ab^^†^	508.6^b^^†^	16.01	0.015	0.001	0.001
Protein, g / wk (6.25*)	124.5	120.8	128.1	120.4	3.29	0.329	0.001	0.001
Fat, g / wk	26.37^c^	29.26^b^	34.49^a^	36.53^a^	0.69	0.001	0.001	0.001
Starch, g / wk	300.1^a^^†⁎^	271.3^a^^†^	269.0^a^*	229.2^b^	8.24	0.001	0.001	0.001
Crude fibre, g / wk	18.1	17.8	19.2	18.2	0.54	0.338	0.001	0.007
ADF, g / wk	23.9	23.0	24.3	22.7	0.62	0.265	0.001	0.001
NDF, g / wk	64.3^a^	60.4^ab^	62.2^ab^	56.1^b^	1.75	0.027	0.001	0.001
Chitin, g / wk	n.a.	1.46	2.92	4.37	-	-	-	-
Crude ash, g / wk	28.3	27.0	28.2	26.2	0.73	0.158	0.001	0.001
Ca, g /wk	4.29	4.19	4.47	4.30	0.103	0.303	0.001	0.001
P, g / wk	3.10	2.98	3.13	2.91	0.083	0.218	0.001	0.001
Mg, g / wk	1.18	1.14	1.20	1.13	0.032	0.314	0.001	0.001
ME, MJ / wk	8.17^a^	7.70^ab^	7.95^ab^	7.19^b^	0.22	0.035	0.001	0.001
CP:ME ratio	15.8^d^	16.2^c^	16.7^b^	17.2^a^	0.025	0.001	0.001	0.001
Growth performance								
Initial weight, g	41.9	41.7	41.5	41.7	0.157	0.488	-	-
BW, g	1,118	1,037	1,073	981	49.2	0.280	0.001	0.033
CV of BW, %	20.0^b^^†^	26.0^ab^	27.0^ab^^†^	33.9^a^	2.34	0.006	0.001	0.129
Nutrient and energy conversion ratios								
FCR-1, FMI / BWG	1.59^b^	1.67^b^	1.69^b^	1.87^a^	0.034	0.001	0.001	0.102
FCR-2, DMI / BWG	1.414	1.398	1.332	1.364	0.024	0.132	0.001	0.022
PCR, g CP / 100 g BWG	30.5^a^	31.2^ab^	30.8^ab^^†^	33.0^b^^†^	0.578	0.030	0.001	0.046
ECR, MJ ME / 100 g BWG	1.948	1.939	1.859	1.92	0.112	0.306	0.001	0.022

Abbreviations: Ca, calcium; BW, body weight; BWG, body weight gain; ECR, energy conversion ratio (i.e., MJ metabolizable energy needed to gain 100 g BW); FCR-1, feed conversion ratio based on FMI (i.e., g FM intake per g BW gain); FCR-2, feed conversion ratio corrected for DM intake (i.e., g DM intake per g BW gain); P, phosphorus; PCR, protein conversion ratio (i.e., g protein needed to gain 100 g BW).

a-d Groups denoted with different letters differ significantly (Tukey, *P* < 0.05). The signs (†,*) indicate a tendency to differ (Tukey, 0.05 < *P* ≤ 0.10). Data are presented as LSM and their SE.

1 Dietary treatment groups: ad-libitum feed without access to BSFL (CON), or with BSFL amounting to 10% (L10), 20% (L20), or 30% (L30) of the feed intake of CON birds. Total number of observations used for statistical analyses, N = 144 (4 treatments each with 6 replicate pens repeatedly measured over 6 weeks); Number of birds, n = 63 per treatment group.

2 G = group effect, W = time effect (week), G × W = group by time interaction.

**Figure 3:**
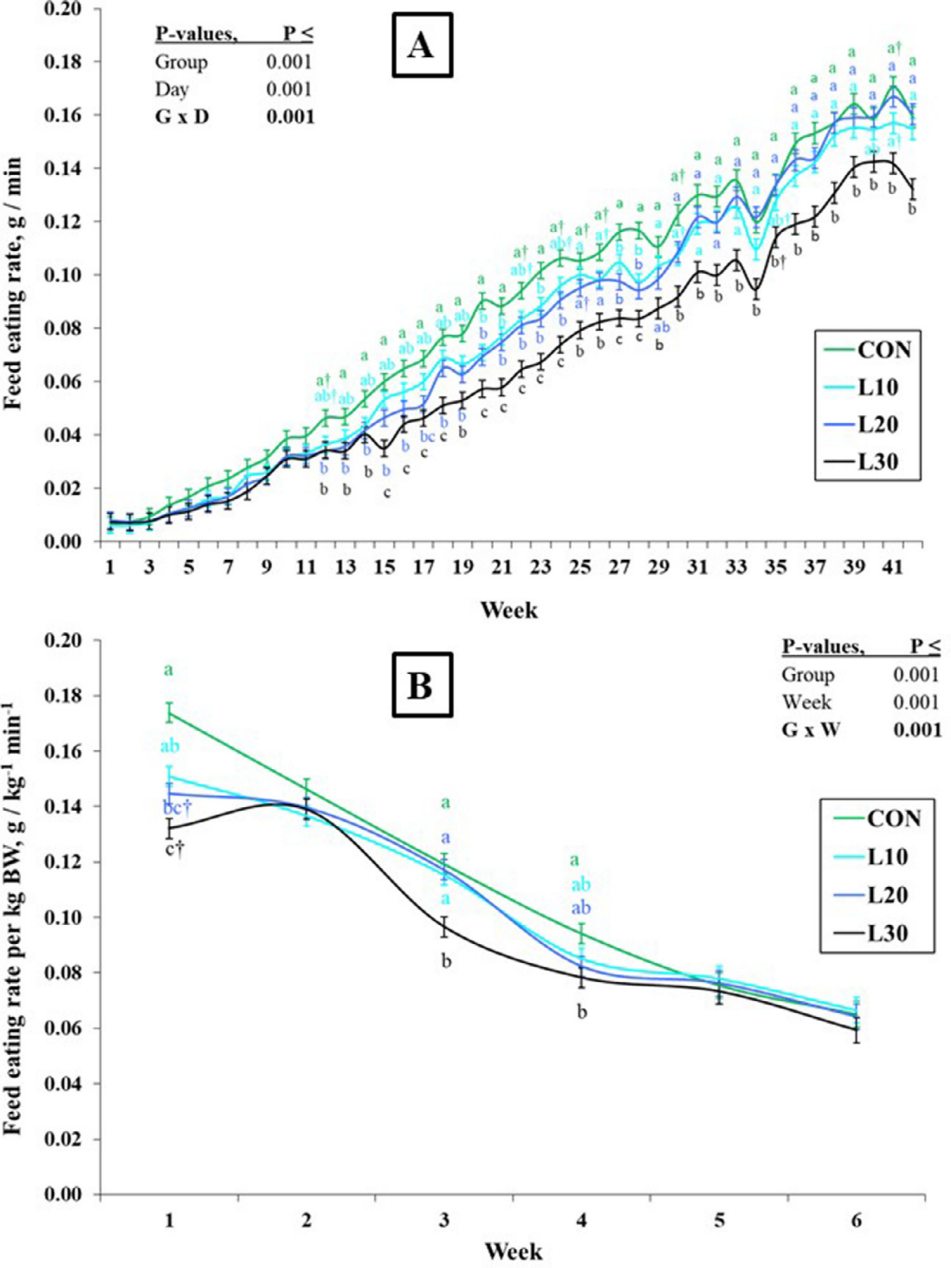
Effects of increasing levels of whole black soldier fly larvae in broiler rations on feed eating rates without (FER) (A) or with an adjustment per kg body weight (FER_BW) (B). Values are LSM with their SE. a-c: Values denoted with different letters at the same point within each panel differ significantly (Tukey, *P* < 0.05). The symbol † indicates a tendency of two treatments to differ (Tukey, 0.05 < *P* ≤ 0.10).

Consistent with the FI pattern, the FER of the birds (g / min) was lower in L10 to L30 than at CON (*P* < 0.001; Table 2), increased linearly in all groups, and became more clearly BSFL-dose-dependent over time (Figure 3A). The increase in FER of the birds from the first to the last d of experiment was about 25-fold. Adjusted for BW, the feed eating rate (FER_BW) showed a linear decrease in all 4 groups (Figure 3B), although differences among the groups were also strongly dependent on time. While CON birds had a higher FER_BW than L30 birds at wk 1, 3, and 4 (*P* < 0.05), there was no difference between groups by wk 5 (*P* > 0.05).

**Figure 4:**
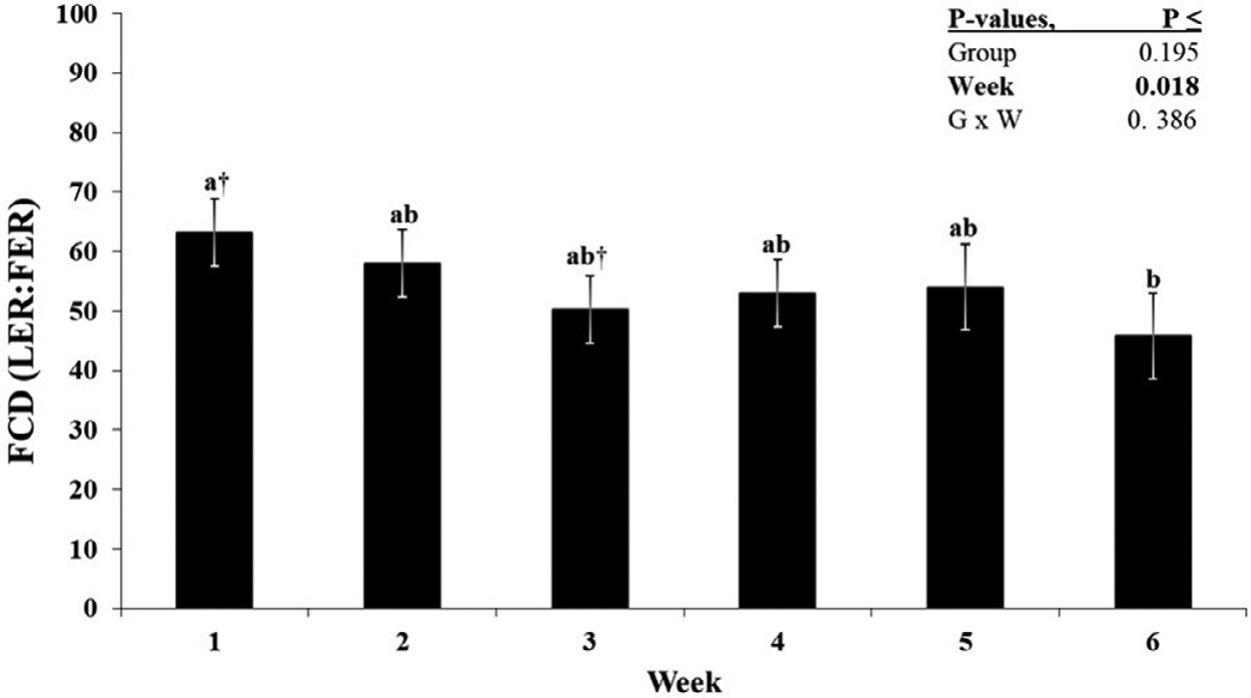
Time dependent changes of fold change differences (FCD) in ratios of larvae eating rate to feed eating rate (LER:FER) of broilers fed increasing levels of BSFL as part of their rations. Bars are LSM with their SE. For overall average group LSM see Table 2. a-b: Time points denoted with different letters differ significantly (Tukey, *P* < 0.05). The sign (†) indicates a tendency to differ (Tukey, 0.05 < *P* ≤ 0.10).

In order to compare time-dependent changes in larvae and feed eating rates simultaneously over 6 wk, we calculated FCD in ratios of LER to FER only in the 3 larvae consuming groups. The FCD was not influenced by the amount of larvae offered to the birds (*P* = 0.195; Table 2), but slightly decreased by time with a difference only between wk 1 and wk 6 (*P* = 0.018; Figure 4). The FCD in BSFL to feed eating rates provided almost the same results when BSFL and feed eating rates were adjusted for BW (see Table 2).

### Nutrient and Energy Intakes, Growth Performance, and Feed Conversion Indices

In L30 birds the CP intake was lower than in CON birds at wk 3 (*P* < 0.05; Supplementary Figure 1A), while L20 birds had a higher CP intake than CON and L10 birds at wk 6 (*P* < 0.05). The average fat intake was higher in L30 and L20 birds than in CON and L10 birds (*P* < 0.05, Table 3). Higher fat intake in L30 birds than in CON birds occurred for the first time at wk 2 (*P* < 0.05; Supplementary Figure 1B), which then became more pronounced in subsequent weeks. By wk 4, L10 birds also consumed a higher amount of fat than did the CON birds (*P* < 0.05).

In line with FI, the average starch and NDF consumption was lower in L30 than in CON birds (*P* < 0.05; Table 3). The average ME intake of L30 birds was lower as compared to that of CON (*P* < 0.05) with time-dependent differences at wk 3 and 4 (Supplementary Figure 1C). Also, ME intake of L30 birds was lower than that of L20 birds at wk 5 and 6 (*P* < 0.05; Supplementary Figure 1C). Provision of BSFL increased the CP:ME ratio in a linear fashion with differences among all 4 groups (Table 3), implying less energy availability per unit protein consumed with increasing levels of BSFL in the ration. The average CF and ADF intake did not differ among the groups (*P* > 0.05, Table 3), while time-dependent differences indicated higher CF and ADF intakes in L20 than in CON at wk 6 (data not shown). Significant time-dependent differences were also quantified for crude ash and mineral (e.g., Ca, P) intake. As shown in Supplementary Figure 2A, both L20 and L30 groups had higher Ca intake than L10 and CON at wk 6, whereas P and crude ash intakes were higher only in L20 at wk 6 (Supplementary Figures 2B and 2C; respectively).

**Figure 5:**
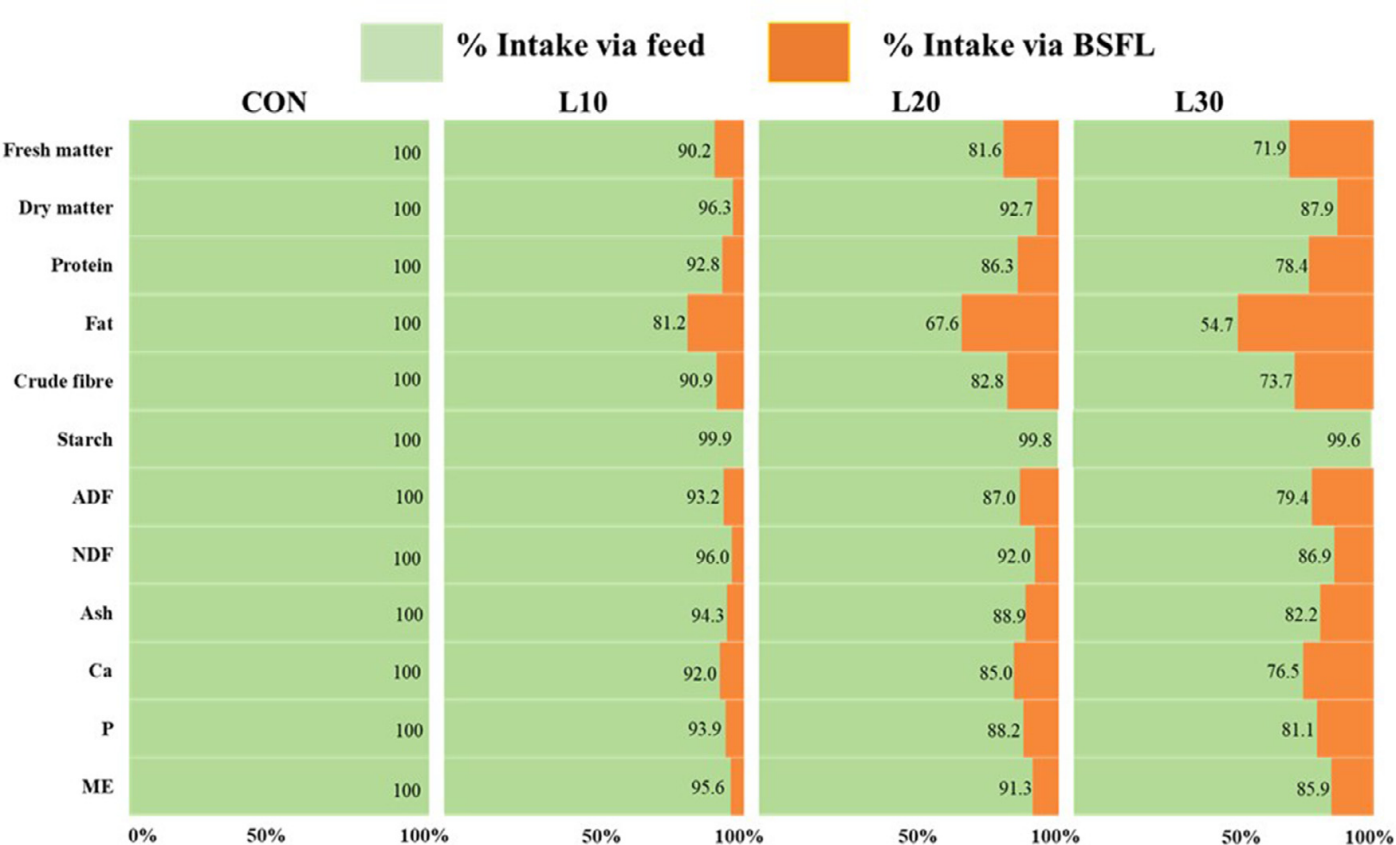
Effects of increasing levels of whole black soldier fly larvae in broiler rations on proportional nutrient and energy intakes via feed and larvae. For exact numbers and further details see Supplementary Table 2.

Although the FMI via BSFL corresponded to the BSFL provision levels (i.e., 10, 20 and 30% of CON feed intake), due to the low DM content of BSFL (i.e., 31.2% DM), the contribution of BSFL intake to relative DMI was smaller than the pre-set levels of BSFL provision (Figure 5). The only nutrient that was exclusively taken from feed in all 4 groups (i.e., 99.6–100%) was starch. With the exception of fat, relative nutrient and energy intakes via BSFL were either approximately similar to or lower than the pre-set BSFL provision levels (i.e., 10, 20 or 30%, respectively; Figure 5). Due to their high fat content the relative fat intake via BSFL consumption was 18.8% in L10, 32.4% in L20 and 45.3% in L30 groups, respectively, which far exceeded the pre-set BSFL provision levels. The relative intakes via feed and BSFL for selected nutrients and ME are shown in Supplementary Table 2.

**Figure 6:**
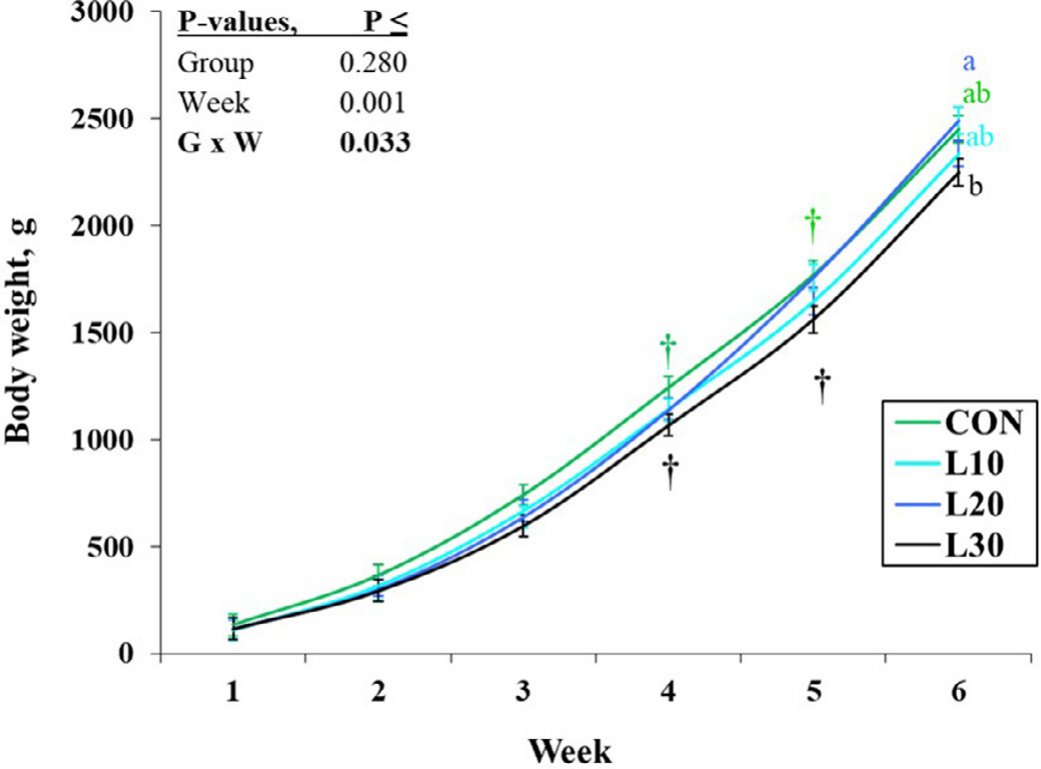
Time-dependent changes in body weight of broilers offered increasing levels of whole black soldier fly larvae in their rations. Values are LSM with their SE. a-b: Values denoted with different letters at the same point differ significantly (Tukey, *P* < 0.05). The symbol † indicates a tendency of two treatments to differ (Tukey, 0.05 < *P* ≤ 0.10).

The provision of BSFL at 30% instead of 20% influenced BW in a time dependent manner. A treatment group by week interaction for BW (*P* = 0.033) indicated a lower BW in L30 than L20 birds at wk 6 (*P* < 0.05; Figure 6), whereas the larvae consuming groups did not differ from the CON group at any time point (*P* > 0.05). Although consumption of BSFL resulted in a gradual increase of heterogeneity in BW in a dose-dependent manner (Table 3), a significant increase in CV of BW was observed only in L30 as compared to CON groups (*P* < 0.05; Table 3).The L20 birds also tended to have increased CV of BW when compared with CON (*P* < 0.10), whereas CV of BW did not differ between L10 and CON groups (*P* > 0.05).

The overall feed conversion rate based on FMI (i.e., FCR-1) was higher in L30 than in the other groups (*P* < 0.05, Table 3). The overall FCR-2 based on DMI did not differ among the groups (*P* = 0.132, Table 3), whereas time-dependent differences indicated that the FCR-2 was lower in L30 than in CON birds at wk 4, but at wk 5 it was lower in L20 than those in L10 and L30 birds (*P* < 0.05, Supplementary Figure 3A). Overall, in the L30 group PCR was higher as compared with the CON group (*P* < 0.05; Table 3), which was due to time-dependent increases at wk 1, 2, and 5 (*P* < 0.05; Supplementary Figure 3B). At wk 5, ECR was lower in L20 than in L10 and L30 groups (Supplementary Figure 3C).

### Potential Trade-Offs in Nutrient and Energy Intakes

The 4 treatment groups exhibited a distinct clustering of the selected nutrient intake patterns, representing energy, protein, fiber, and mineral intakes (Supplementary Figure 4). Despite the slight differences from week-to-week, a gradual differentiation of the groups was repeatedly observed over 6 wk, corresponding to the increasing levels of BSFL in the ration.

The CP, NDF, starch, and crude ash intakes were positively correlated with each other (see loadings in Supplementary Figure 4, right panels) and they contributed to the higher variation observed on Component 1 (Supplementary Figure 4; see X-axis in all left-panels). The main driving force of the differentiations among the 4 treatment groups was however the crude fat intake as it was less correlated with other nutrients and always corresponded well to the direction of the differentiation (see loadings in Supplementary Figure 4- right panels).

### Blood Metabolites and Immunoglobulin Isotypes

Plasma albumin, globulin and total protein concentrations were not affected by the dietary treatments (*P* > 0.05; Table 4). In the L30 group plasma UA concentration was higher than in the CON group (*P* < 0.05) although in the L20 group birds tended to show higher UA (*P* = 0.052) as well. Similar to UA, ALP activity levels also increased with increasing levels of BSFL in the ration, with L20 and L30 groups showing a higher serum ALP concentrations than in CON (*P* < 0.05; Table 4). In addition, ALP levels tended to increase in L30 birds when compared with L10 (*P* < 0.10). The level of BSFL in the ration had no effect on plasma IgY and IgM concentration of the chickens (Table 4), whereas L30 birds tended to have higher plasma IgA concentration than did L10 birds (*P* = 0.053).

**Table 4:** Effects of increasing levels of whole black soldier fly larvae in broiler rations on selected blood metabolites and immunoglobulin concentrations of broilers.

	Dietary treatment groups^1^					*P*-values^2^, ≤		
	CON	L10	L20	L30	SE	G	W	G × W
Metabolites								
Albumin, g / l	11.20	11.69	11.51	11.60	0.181	0.199	0.001	0.670
Globulin, mmol / l	14.75	14.48	14.64	14.43	0.457	0.952	0.370	0.140
Total protein, g / l	25.47	26.34	26.40	26.23	0.497	0.432	0.003	0.764
Uric acid, µmol / l	375.0^b^^†^	410.9^ab^	485.2^ab^^†^	498.9^a^	30.61	0.017	0.002	0.820
ALP^3^, U / l	1,757^b^	2,266^ab^^†^	3,629^a^	4,014^a^^†^	517	0.009	0.001	0.079
Immunoglobulins								
IgY, mg / mL	1.60	1.67	1.56	1.56	0.176	0.966	0.002	0.569
IgM, mg / mL	0.145	0.153	0.139	0.163	0.010	0.288	0.001	0.560
IgA, mg / mL	0.191	0.185^†^	0.228	0.260^†^	0.021	0.053	0.155	0.148

Abbreviation: ALP, alkaline phosphatase (3measured in serum).

a-b Groups denoted with different letters differ significantly (Tukey, *P* < 0.05). The sign (†) indicates tendency of two diets to differ (Tukey, 0.05 < *P* ≤ 0.10).

1 Dietary treatments: ad-libitum feed without access to BSFL (CON), or with BSFL amounting to 10% (L10), 20% (L20), or 30% (L30) of the feed intake of CON birds. Total number of observations used for statistical analyses, N = 96 (i.e., 2 birds sampled from each of 6 pens allocated to each of 4 treatments at weeks 4 and 6).

2 G: group effect, W = time effect for weeks 4 and 6, G × W = treatment by week interaction.

## DISCUSSION

In this study, we first assessed apparent interest of broilers in eating BSFL when offered at either 10, 20, or 30% of voluntary FI of CON chickens that received no BSFL. Next, nutrient and energy intakes of the birds through larvae and voluntary feed consumption were estimated, and their growth performance, nutrient conversion efficiency, plasma metabolites, and immunoglobulins concentrations were then assessed. Larvae eating time and eating rates of broilers indicated a strong preference for BSFL over regular feed. We found that chickens can consume up to 30% of their voluntary FI as BSFL in just a few minutes after a short (3 d) learning period. Apparent interest of chickens in BSFL as compared to regular feed was at least 50 times higher, implying a great potential of BSFL not only to be included in broiler rations, but also as an edible environmental enrichment tool. The nutrient and energy intakes of broilers by consumption of feed and larvae and their utilization indices for growth indicate that feeding unprocessed whole BSFL up to 20% of voluntary FI has no adverse effects. A higher level of BSFL in the daily ration (i.e., 30%) lead to a lower total DMI despite of an elevated FMI, and eventually resulted in lower ME intake that could not be compensated through the high fat content of BSFL (28% fat in DM), which, on the other hand might have impaired DMI of the birds. The lower overall energy intake in L30 birds was likely the main reason for their lower BW compared to L20 birds at wk 6. Equally important, provision of BSFL at 30% of FI in the ration leads to a lower protein utilization efficiency, likely due to a lower protein:energy intake ratio leading to higher nitrogen excretion as suggested by the higher plasma uric acid concentration in L30 birds. In addition, the higher fat intake in L20 and L30 groups might likely affect liver function and subsequently increased ALP concentration in L30.

### Larvae Eating Time and Eating Rate

After a short (3 d) learning period, the birds in the larvae consuming groups were able to consume their daily BSFL portions just in a few minutes. Except for the first day, there was no difference in TSL among larvae fed groups. The lack of differences in TSL among groups consuming larvae might be largely related to within group variation (see error bars on Figure 1A). However, larvae eating rate depended on the amount of BSFL offered to the birds, with L10 birds eating more larvae per min than L20 and L30 birds. The higher LER could be due to increased larval feeding competition in L10 birds as a result of fewer palatable nutrients such as fat and protein, which are known to be perceived by chickens (Cheled-Shoval et al., 2017). We quantified a strong linear increase in LER with time by more than 200-fold from the first day to the last day of experiment, indicating a steadily increasing eating rate in response to time. Increasing LER over time was considered to be a result of two interrelated factors, social learning and growth, that is, increasing body weight (Slagsvold and Wiebe, 2011; Tallentire et al., 2018). In order to separate, at least partly, the impact of these 2 factors on larvae eating rate, we adjusted LER for BW of the birds and plotted it against time. After adjustment for BW, the larvae eating rate showed a completely different pattern, decreasing approximately 3-fold from the first to the last week, but did not depend on the amount of BSFL offered to the birds. Combined with the sharply decreasing TSL during the first 3 d, the linearly decreasing LER over time may indicate that the birds likely learn to eat BSFL already during the first week, but that their motivation to consume larvae in proportion to their body mass declines to some extent over time.

A previous study found that young birds take longer to learn foraging behaviors than adults, indicating that learning strategies change with age (Franks and Thorogood, 2018). However, there is a gap in knowledge regarding how long it takes for broiler chickens to learn foraging behavior, especially if there is competition for limited feed resources, which is usually not the case because feed is regularly offered to birds ad libitum.

The eating rate for the regular feed (**FER**) increased from wk 1 to wk 6 by about 25-fold in CON, and after adjustment for BW, FER_BW declined over time. In order to compare time-dependent changes in larvae and feed eating rates over 6 wk in the 3 broiler groups offered larvae, we calculated FCD as ratios of LER to FER. The ratio of larval eating rate to foraging rate (i.e., FCD) decreased from about 60-fold to 50-fold from the first week to the last week, implying a nearly constant preference of at least 50-fold in favor of larval eating over regular feed. The high interest, that is, preference of chickens for BSFL compared to regular feed opens the possibility of including whole BSFL in daily rations for broilers. In addition, it also suggests a great potential of BSFL as an edible environmental enrichment when used in small amounts to stimulate the birds. Nevertheless, there is a lack of knowledge about the palatability of whole BSFL for poultry. Insect larvae are rich in various nutrients and are one of the natural feed sources of poultry, which are very motivating for consumption (Bokkers and Koene, 2002), so birds may clearly prefer larvae over regular feed. Cullere et al. (2016) evaluated BSFL meal as a dietary supplement for quail in a feed selection trial and found that quail preferred the 15% BSFL meal diet 53.8% compared to a 44.1% preference for the control diet, suggesting that poultry favor BSFL over regular feed. Ipema et al. (2020b) also observed a strong appetite value of live BSFL for broilers which was associated with a higher activity and increased foraging behavior. Furthermore, chickens show a clear preference for insect larvae meal (e.g., *Tenebrio molitor*) as compared to classical protein feedstuff, that is, extruded semi-whole soybean meal (Nascimento Filho et al., 2020). Previous studies suggested that providing mealworms or BSFL in broiler diets promotes animal welfare by facilitating natural behavior and reducing anxiety, increasing activity, and decreasing the incidence of leg problems (Pichova et al., 2016; Ipema et al., 2020a; Ipema et al., 2020b). When the birds in our study were offered with the predetermined BSFL portions in the morning, they stopped eating regular feed until the larvae on the plate were completely eaten, with some evidence of competitive behavior (Supplementary video 1). Accordingly, voluntary intake of regular feed was lower in larvae fed groups than in the CON groups, and the lower voluntary FI in L30 than in L10 birds suggests a BSFL-dose-dependent nutrient and energy intake via regular feed.

### Animal Health, Nutrient Intakes, and Growth Performance

As reported earlier (Cullere et al., 2016; Kawasaki et al., 2019) in our study BSFL feeding was not associated with mortality, metabolic disorders, wet litter, or foot and leg problems. The nutrient composition of the BSFL used was in the range of what was previously observed (Makkar et al., 2014; Liland et al., 2017; Moula et al., 2018). In the current study, 30% of BSFL in the ration led to a lower total DMI which is in line with earlier observations (Ipema et al., 2020b). Despite the increased FMI of the L30 birds in the last 2 wk of the experiment, their DMI was the lowest, resulting in a lower ME intake that could not be compensated by the higher fat intake via BSFL consumption. The results of the PCA analysis suggest that the higher fat intake in L30 birds was the main driving force leading to their lower DMI. It is known that high levels of dietary fat alters the response of hypothalamic appetite-related peptides (Wang et al., 2017), which is associated with circulating insulin (Obrosova et al., 2007). The physical constrains in digestive tract capacity of broilers might limited FMI regulation in association with energy intake (Dozier III et al., 2006; Brickett et al., 2007).

Previous studies showed that BSFL meal consumption at 5% of dietary DM increased broiler growth (Lee et al., 2018). Similarly, Biasato et al. (2020) observed positive effects of dietary inclusion of partially defatted BSFL-meal as partial replacement of soybean meal, corn gluten meal, and soybean oil at low inclusion levels (i.e., 5%) on cecal microbiota or the gut mucin dynamics. However, they reported a negative influence of high inclusion levels (i.e., 15%) such as a partial reduction of microbial complexity and reduction of potentially beneficial bacteria. Józefiak et al. (2018) reported positive influence of using full-fat insect meals (*Hermetia illucens, Gryllodes sigillatus, Shelfordella lateralis, Gryllus assimilis*, and *Tenebrio molitor*) in low amounts (i.e., 0.05–0.2%) on the top of broiler diets in terms of modulating microbial populations in the gastrointestinal tract. Results of a meta-analysis study suggested that partially substitution of conventional protein sources (i.e., less than 10%) with insects (such as black soldier fly larvae, mealworms, and maggots) in poultry diet has no adverse effect on the growth, FI, and FCR (Moula and Detilleux, 2019). Also, Moula et al. (2018) reported that body weights of chickens fed standard feed supplemented with 8% whole defrozen larvae were higher than those of control chickens. In contrast, we observed a lower BW in L30 compared to L20 birds in wk 6, indicating higher BSFL inclusion levels may affect broiler growth, but none of the BSFL groups differed in BW from the CON group. Ipema et al. (2020b), attributed the lower growth of BSFL-fed chickens to an imbalance in amino acid (**AA**) uptake. However, despite the large differences in crude protein contents, we did not find large differences in the composition of essential AA of age-specific diets and BSFL (Supplementary Table 1). It is likely that in our study overall protein digestibility might have been affected in the L30 group because the BSFL contained a relatively high chitin content (73 g/kg DM). It was earlier suspected by Dabbou et al. (2018) that reduced growth of broilers fed defatted BSFL meal could be due to the chitin content of BSFL which might negatively influence the protein digestibility. It should be noted that per design of the experiment the final rations of the groups were not isocaloric or isonitrogenous after feeding different amounts of BSFL in addition to the complete diets, which might have contributed to differences in nutrient intakes and growth performance of the birds.

### Blood Metabolites and Immunoglobulin Isotypes

In chickens, UA is the main end product of nitrogen metabolism (Donsbough et al., 2010), and excessive protein consumption can lead to an increased blood UA concentration (Musigwa et al., 2020). We observed a linear increase of the CP:ME ratio in response to feeding increasing levels of BSFL which was accompanied by a numerical increase of plasma UA concentration, a higher plasma UA concentration in L30 birds while protein utilization was also less efficient in the same birds. Similar to UA, serum ALP activity levels increased with rising levels of BSFL in the ration, with the L20 and L30 groups having higher serum ALP concentrations than CON. The ALP enzyme is involved in active bone formation, and in chickens it is considered as a marker for skeletal health, bone disease and liver damage (Jiang et al., 2013; Senanayake et al., 2015). The elevated serum ALP concentration in L30 birds might be explained with the higher fat intake (see section 3.2) which was possibly associated with adverse effects on liver function (Jiang et al., 2013). The plasma IgY and IgM concentrations were not consistently affected by dietary treatment, however, the L30 birds tended to have a higher plasma IgA concentration than those in L10 group. Increased IgA concentration in response to feeding BSFL may suggest an activation of the humoral immune system (Song et al., 2021). However, information on the effects of feeding whole BSFL to broiler chickens on blood immunoglobulin concentrations is lacking. Nevertheless, El-Hack et al. (2020) discussed that the improved immune function in BSFL-fed hens may be due to chitin content in BSFL meal, which has the ability to stimulate the immune system.

## CONCLUSIONS

We conclude that chickens can consume BSFL up to 30% of their voluntary FI in a few minutes after a short period of learning. Larvae eating time and eating rates of broilers suggest a strong preference for BSFL over regular feed. Whole BSFL can be included in broiler rations up to 20% without adverse effects on growth performance and nutrient conversion efficiency, whereas higher levels are associated with lower protein utilization efficiency, possibly due to lower total energy intake despite the high fat content of BSFL.

## Appendix Supplementary materials

**Supplementary Figure 1.** Effects of increasing levels of whole black soldier fly larvae in broiler rations on crude protein intake (A), crude fat intake (B) and metabolizable energy intake (C) in broilers during the experimental weeks. Values are LSM with their SE. a-c: Values denoted with different letters at the same point within each panel differ significantly (Tukey, P < 0.05). The symbol † indicates a tendency of two treatments to differ (Tukey, 0.05 < P ≤ 0.10). Image, application 1

**Supplementary Figure 2.** Effects of increasing levels of whole black soldier fly larvae in broiler rations on calcium intake (A), phosphorus intake (B) and ash intake (C) in broilers during the experimental weeks. Values are LSM with their SE. a-c: Values denoted with different letters at the same point within each panel differ significantly (Tukey, P < 0.05). The symbol † indicates a tendency of two treatments to differ (Tukey, 0.05 < P ≤ 0.10). Image, application 2

**Supplementary Figure 3.** Effects of increasing levels of whole black soldier fly larvae in broiler rations on FCR-DM (A), protein conversion ratio (B) and energy conversion ratio (C) in broilers during the experimental weeks. Values are LSM with their SE. Image, application 3

**Supplementary Figure 4.** Principle components analysis (left panels) to identify which nutrients are the driving forces in differentiation of experimental groups over 6 experimental weeks. The component 1 and component 2 represent the samples variance. Ride side panels present the loadings of the principal components analysis. The X-axis and Y-axis values represent the contributing weights of each nutrient to the principal components 1 and 2 of the PCA component, respectively. Variables with loadings showing the same direction are highly correlated. All variables had the same units of measurement (i.e. g/wk). Abbreviations: **CFI_T**: crude fat intake; **CPI_T**: crude protein intake; **CashI_T**; crude ash intake; **CNDFI_T**; NDF intake; **starch_T**: starch intake. Image, application 4

Image, application 5

**Supplementary Video 1.** A short video showing the high interest of chickens for consuming black soldier fly larvae with some evidence of competitive behavior. Image, video 6
