# Supplementary material for: Effects of increasing levels of whole Black Soldier Fly (Hermetia illucens) larvae in broiler rations on acceptance, nutrient and energy intakes and utilization, and growth performance of broilers
Source: Poult Sci. 2022 Sep 24;101(12):102202. doi: 10.1016/j.psj.2022.102202 (PMC9579412; doi:10.1016/j.psj.2022.102202)
Supplement: Supplementary file 1 — Supplementary Figure 1. Effects of increasing levels of whole black soldier fly larvae in broiler rations on crude protein intake (A), crude fat intake (B) and metabolizable energy intake (C) in broilers during the experimental weeks. Values are LSM with their SE. a-c: Values denoted with different letters at the same point within each panel differ significantly (Tukey, P < 0.05). The symbol † indicates a tendency of two treatments to differ (Tukey, 0.05 < P ≤ 0.10). [file mmc1.pptx]

## Slide 1
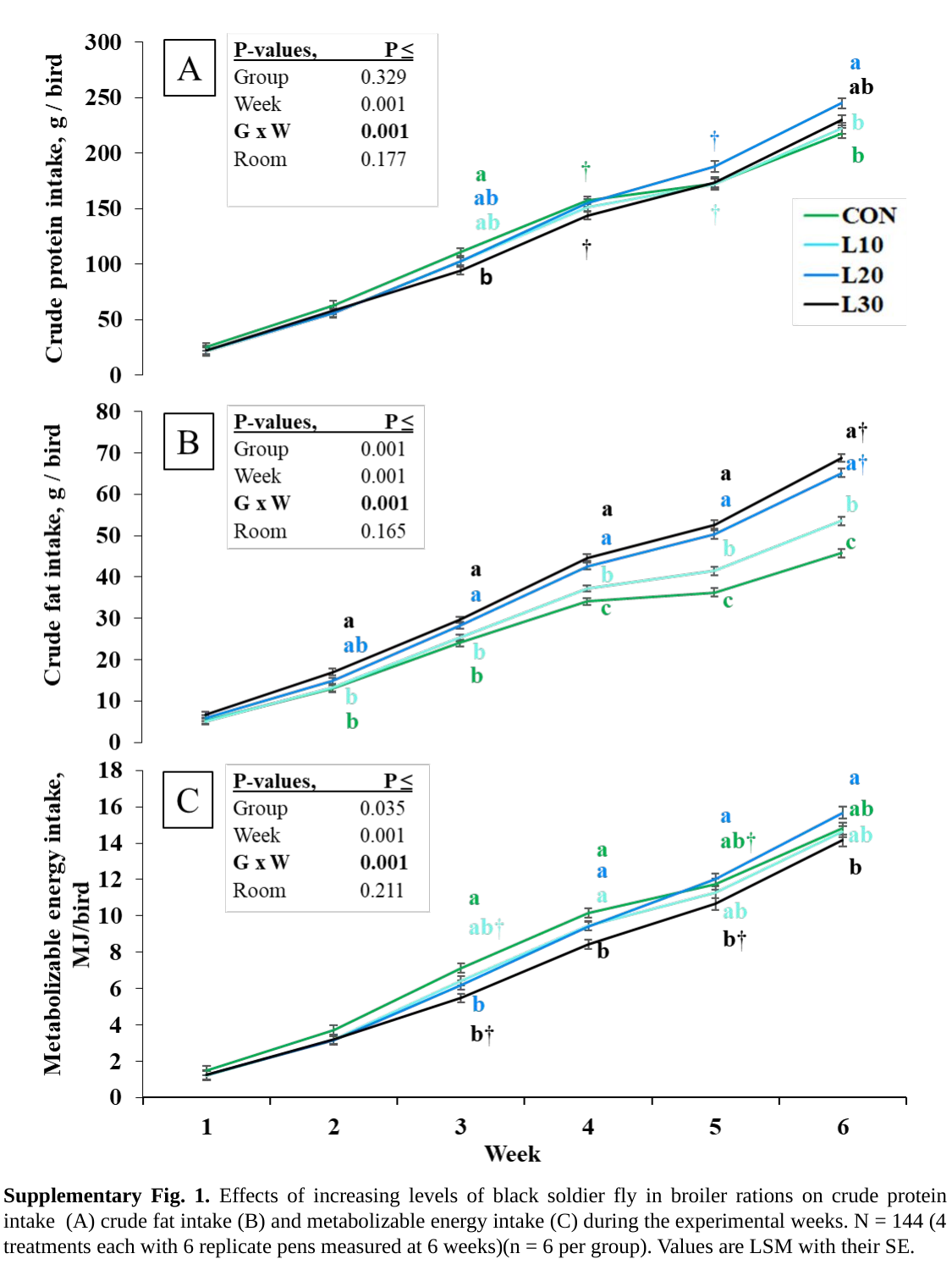

Supplementary Fig. 1. Effects of increasing levels of black soldier fly in broiler rations on crude protein intake (A) crude fat intake (B) and metabolizable energy intake (C) during the experimental weeks. N = 144 (4 treatments each with 6 replicate pens measured at 6 weeks)(n = 6 per group). Values are LSM with their SE.
