# Supplementary material for: Effects of increasing levels of whole Black Soldier Fly (Hermetia illucens) larvae in broiler rations on acceptance, nutrient and energy intakes and utilization, and growth performance of broilers
Source: Poult Sci. 2022 Sep 24;101(12):102202. doi: 10.1016/j.psj.2022.102202 (PMC9579412; doi:10.1016/j.psj.2022.102202)
Supplement: Supplementary file 3 — Supplementary Figure 3. Effects of increasing levels of whole black soldier fly larvae in broiler rations on FCR-DM (A), protein conversion ratio (B) and energy conversion ratio (C) in broilers during the experimental weeks. Values are LSM with their SE. [file mmc3.pptx]

## Slide 1
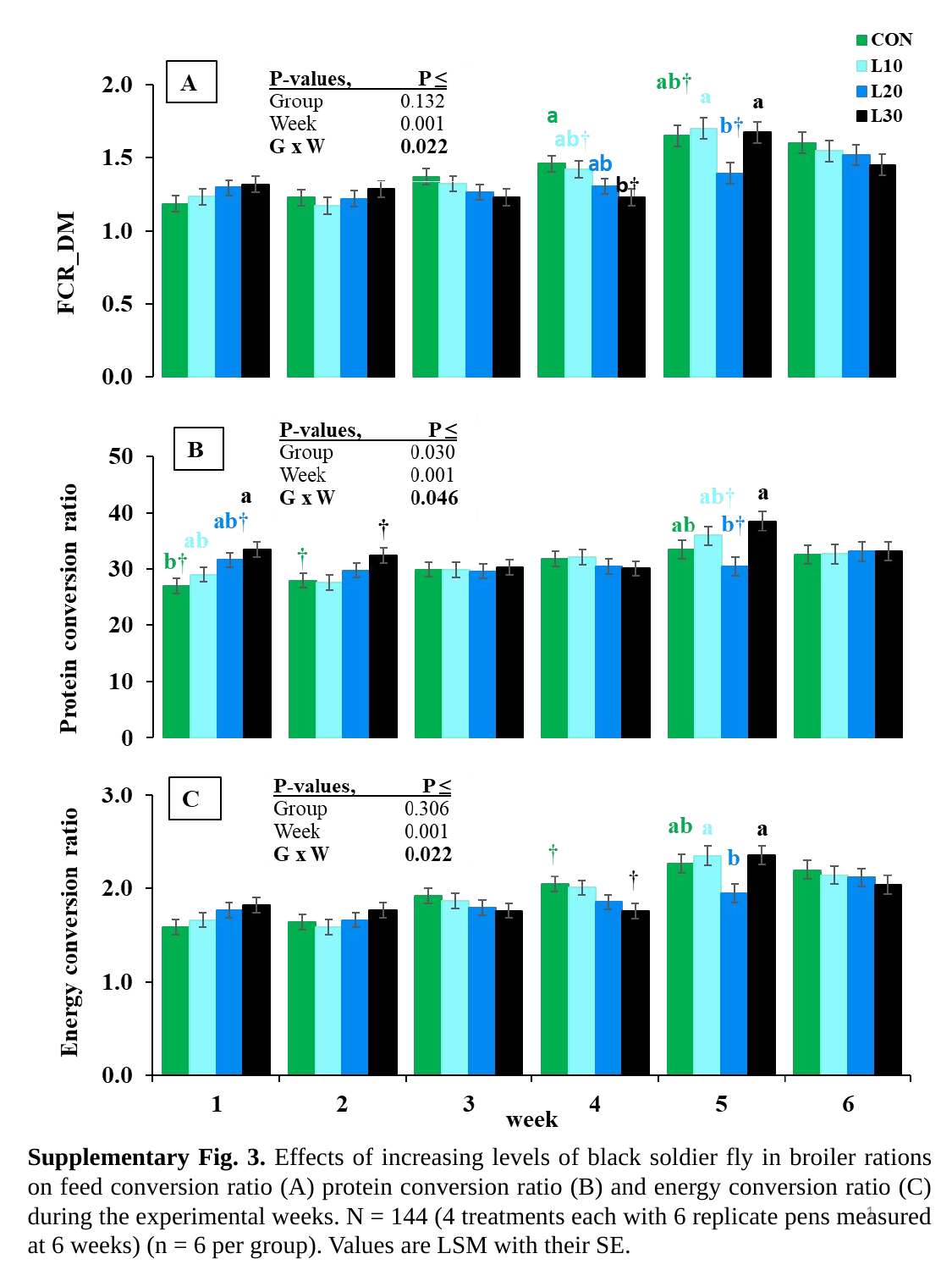

Supplementary Fig. 3. Effects of increasing levels of black soldier fly in broiler rations on feed conversion ratio (A) protein conversion ratio (B) and energy conversion ratio (C) during the experimental weeks. N = 144 (4 treatments each with 6 replicate pens measured at 6 weeks) (n = 6 per group). Values are LSM with their SE.
1
