# Supplementary material for: Effects of increasing levels of whole Black Soldier Fly (Hermetia illucens) larvae in broiler rations on acceptance, nutrient and energy intakes and utilization, and growth performance of broilers
Source: Poult Sci. 2022 Sep 24;101(12):102202. doi: 10.1016/j.psj.2022.102202 (PMC9579412; doi:10.1016/j.psj.2022.102202)
Supplement: Supplementary file 4 — Supplementary Figure 4. Principle components analysis (left panels) to identify which nutrients are the driving forces in differentiation of experimental groups over 6 experimental weeks. The component 1 and component 2 represent the samples variance. Ride side panels present the loadings of the principal components analysis. The X-axis and Y-axis values represent the contributing weights of each nutrient to the principal components 1 and 2 of the PCA component, respectively. Variables with loadings showing the same direction are highly correlated. All variables had the same units of measurement (i.e. g/wk). Abbreviations: CFI_T: crude fat intake; CPI_T: crude protein intake; CashI_T; crude ash intake; CNDFI_T; NDF intake; starch_T: starch intake. [file mmc4.pptx]

## Slide 1
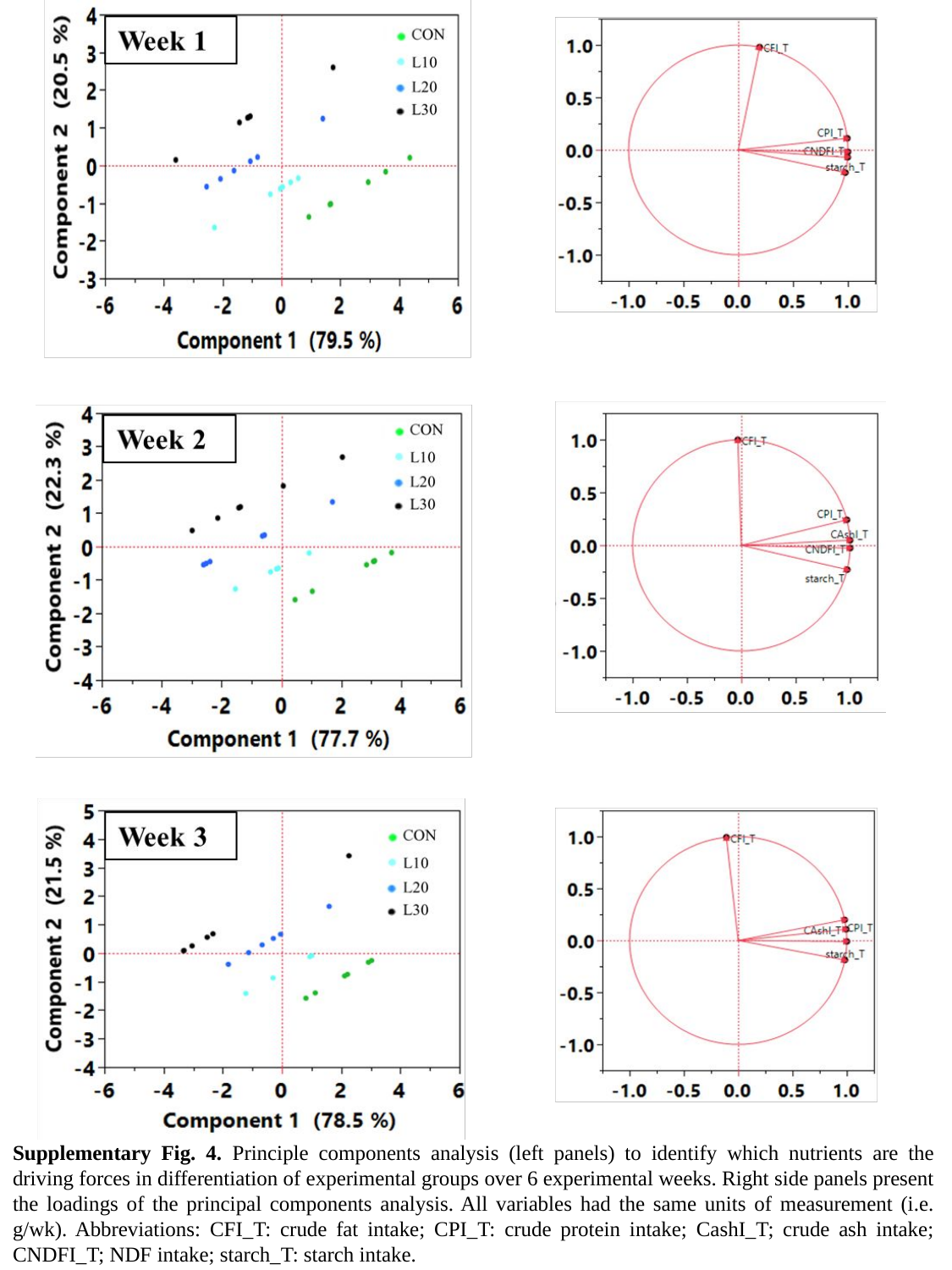

Supplementary Fig. 4. Principle components analysis (left panels) to identify which nutrients are the driving forces in differentiation of experimental groups over 6 experimental weeks. Right side panels present the loadings of the principal components analysis. All variables had the same units of measurement (i.e. g/wk). Abbreviations: CFI_T: crude fat intake; CPI_T: crude protein intake; CashI_T; crude ash intake; CNDFI_T; NDF intake; starch_T: starch intake.

## Slide 2
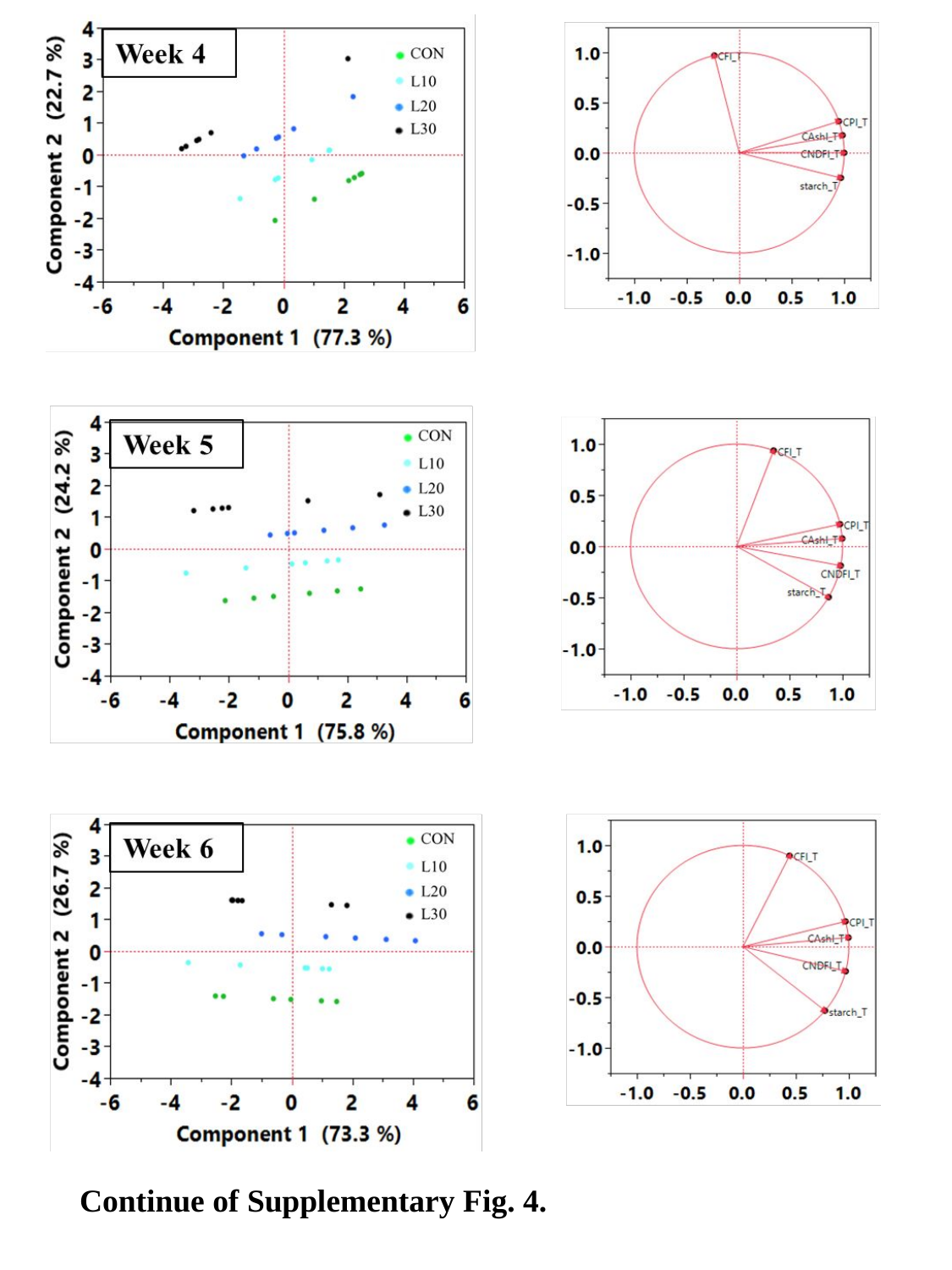

Continue of Supplementary Fig. 4.
