# Supplementary material for: Effects of increasing levels of whole Black Soldier Fly (Hermetia illucens) larvae in broiler rations on acceptance, nutrient and energy intakes and utilization, and growth performance of broilers
Source: Poult Sci. 2022 Sep 24;101(12):102202. doi: 10.1016/j.psj.2022.102202 (PMC9579412; doi:10.1016/j.psj.2022.102202)
Supplement: Supplementary file 5 [file mmc5.docx]

**Supplementary Table 1.** Amino acid (AA) composition (as mg / g DM) of the age-specific basal diets and black soldier fly larvae (BSFL) offered to broilers during 42 experimental days.

| **AA (mg / g DM)** | **Basal diets** | | | **BSFL**  **(d 1-42)** |
| --- | --- | --- | --- | --- |
|  | **Starter**  **(d 1-14)** | **Grower**  **(d 15-28)** | **Finisher**  **(d 29-42)** |  |
| **Essential** |  |  |  |  |
| Arginine | 15.5 | 13.9 | 13.9 | 18.6 |
| Histidine | 6.3 | 6.0 | 6.4 | 12.3 |
| Isoleucine | 9.1 | 8.6 | 8.4 | 14.9 |
| Leucine | 18.9 | 18.2 | 17.8 | 25.6 |
| Lysine | 8.9 | 10.2 | 5.8 | 12.0 |
| Methionine^1^ | 1.2 | 1.3 | 3.7 | 4.8 |
| Phenylalanine | 11.9 | 11.3 | 11.2 | 15.2 |
| Threonine | 9.3 | 8.6 | 8.5 | 15.1 |
| Valine | 9.8 | 9.4 | 9.1 | 21.0 |
| **Non- essential** |  |  |  |  |
| Aspartic acid + Asparagine | 23.9 | 22.2 | 21.5 | 33.9 |
| Glutamic acid + Glutamine | 52.0 | 50.9 | 48.5 | 44.0 |
| Cysteine^1^ | 1.3 | 1.3 | 1.2 | 1.4 |
| Serine | 12.4 | 11.5 | 10.9 | 16.7 |
| Glycine | 10.2 | 9.0 | 8.6 | 20.2 |
| Alanine | 10.1 | 9.6 | 9.3 | 27.9 |
| Tyrosine | 8.4 | 8.2 | 8.1 | 24.4 |
| Proline | 17.6 | 22.2 | 16.7 | 31.9 |
| **Sum of FAA** | **4.2** | **3.8** | **4.2** | **28.0** |
| **Sum of protein bound AA** | **222.8** | **218.5** | **205.4** | **311.9** |
| **Sum of free and protein bound AA** | **227.0** | **222.3** | **209.6** | **339.8** |

^1^ Methionine and cysteine were partly oxidized during hydrolysis, and are thus likely underestimated.

**Supplementary Table 2.** Relative nutrient intakes (%) through feed and BSFL consumption in broilers offered either only regular feed (CON) or increasing levels of BSFL (10-30%) in addition to the regular feed.

| ***Relative intakes via feed, %*** | **Dietary treatments^1^** | | | |  | **P-values^2^, ≤** | | |
| --- | --- | --- | --- | --- | --- | --- | --- | --- |
|  | **CON^3^** | **L10** | **L20** | **L30** | SE | **G** | **W** | **G**×**W** |
| Fresh matter | 100 | 90.1^a^ | 81.7^b^ | 72.0^c^ | 0.42 | 0.001 | 0.001 | 0.001 |
| Dry matter | 100 | 96.3^a^ | 92.7^b^ | 88.0^c^ | 0.22 | 0.001 | 0.001 | 0.001 |
| Protein | 100 | 92.8^a^ | 86.4^b^ | 78.4^c^ | 0.35 | 0.001 | 0.001 | 0.001 |
| Fat | 100 | 81.1^a^ | 67.7^b^ | 54.8^c^ | 0.53 | 0.001 | 0.001 | 0.001 |
| ME MJ | 100 | 95.6^a^ | 91.4^b^ | 85.9^c^ | 0.25 | 0.001 | 0.001 | 0.001 |
| Crude fibre | 100 | 90.8^a^ | 82.9^b^ | 73.8^c^ | 0.42 | 0.001 | 0.001 | 0.001 |
| ADF | 100 | 93.2^a^ | 87.1^b^ | 79.5^c^ | 0.33 | 0.001 | 0.001 | 0.001 |
| NDF | 100 | 95.9^a^ | 92.1^b^ | 86.9^c^ | 0.23 | 0.001 | 0.001 | 0.001 |
| Ash | 100 | 94.2^a^ | 89.0^b^ | 82.2^c^ | 0.29 | 0.001 | 0.001 | 0.001 |
| Ca | 100 | 92.0 ^a^ | 85.1^b^ | 76.6^c^ | 0.35 | 0.001 | 0.001 | 0.001 |
| P | 100 | 93.8^a^ | 88.2^b^ | 81.1^c^ | 0.31 | 0.001 | 0.001 | 0.001 |
| Mg | 100 | 93.1^a^ | 86.9^b^ | 79.3^c^ | 0.34 | 0.001 | 0.001 | 0.001 |
| ***Relative intakes via BSFL, %*** | | | | | | | | |
| Fresh matter | 0 | 9.8^c^ | 18.3^b^ | 28.0^a^ | 0.42 | 0.001 | 0.001 | 0.001 |
| Dry matter | 0 | 3.7^c^ | 7.3^b^ | 12.0^a^ | 0.22 | 0.001 | 0.001 | 0.001 |
| Protein | 0 | 7.2^c^ | 13.6^b^ | 21.6^a^ | 0.35 | 0.001 | 0.001 | 0.001 |
| Fat | 0 | 18.9^c^ | 32.3^b^ | 45.2^a^ | 0.53 | 0.001 | 0.001 | 0.001 |
| ME MJ | 0 | 4.4^c^ | 8.6^b^ | 14.1^a^ | 0.25 | 0.001 | 0.001 | 0.001 |
| Crude fibre | 0 | 9.2^c^ | 17.1^b^ | 26.2^a^ | 0.41 | 0.001 | 0.001 | 0.001 |
| ADF | 0 | 6.8^c^ | 12.9^b^ | 20.5^a^ | 0.33 | 0.001 | 0.001 | 0.001 |
| NDF | 0 | 4.1^c^ | 7.9^b^ | 13.1^a^ | 0.23 | 0.001 | 0.001 | 0.001 |
| Chitin | 0 | 100 | 100 | 100 | - | - | - | - |
| Ash | 0 | 5.8^c^ | 11.1^b^ | 17.8^a^ | 0.29 | 0.001 | 0.001 | 0.001 |
| Ca | 0 | 8.0^c^ | 14.9^b^ | 23.4^a^ | 0.35 | 0.001 | 0.001 | 0.001 |
| P | 0 | 6.2^c^ | 11.8^b^ | 18.9^a^ | 0.31 | 0.001 | 0.001 | 0.001 |
| Mg | 0 | 6.9^c^ | 13.1^b^ | 20.7^a^ | 0.34 | 0.001 | 0.001 | 0.001 |

**^1^**Dietary treatments: ad-libitum feed without access to BSFL (CON), or with BSFL amounting to 10% (L10), 20% (L20) or 30% (L30) of the feed intake of CON birds. Total number of observations used for statistical analyses, N = 144 (4 treatments each with 6 replicate pens repeatedly measured over 6 weeks). Number of observations; Number of birds, n=63 per treatment.

**^2^**G = treatment group effect, W = time effect (week), G×T = treatment group by time interaction.

**^3^**Note that CON intakes via feed (100%) and BSFL (0%) show no within group variation per study design, thus statistical comparisons summarized in this table exclude CON, and refer to BSFL consuming groups (L10-L30) only.
